# An Effective Methodology for Diabetes Prediction in the Case of Class Imbalance

**DOI:** 10.3390/bioengineering12010035

**Published:** 2025-01-06

**Authors:** Borislava Toleva, Ivan Atanasov, Ivan Ivanov, Vincent Hooper

**Affiliations:** 1 Faculty of Economics and Business Administration, Sofia University, St. Kl. Ohridski, 1113 Sofia, Bulgaria; vrigazova@uni-sofia.bg (B.T.); iv.atanasov89@gmail.com (I.A.); 2 SP Jain Global School of Management, Academic City, Dubai P.O. Box 502345, United Arab Emirates; vincent.hooper@spjain.org

**Keywords:** class imbalance, classification, cross validation, resample, shuffle

## Abstract

Diabetes causes an increase in the level of blood sugar, which leads to damage to various parts of the human body. Diabetes data are used not only for providing a deeper understanding of the treatment mechanisms but also for predicting the probability that one might become sick. This paper proposes a novel methodology to perform classification in the case of heavy class imbalance, as observed in the PIMA diabetes dataset. The proposed methodology uses two novel steps, namely resampling and random shuffling prior to defining the classification model. The methodology is tested with two versions of cross validation that are appropriate in cases of class imbalance—k-fold cross validation and stratified k-fold cross validation. Our findings suggest that when having imbalanced data, shuffling the data randomly prior to a train/test split can help improve estimation metrics. Our methodology can outperform existing machine learning algorithms and complex deep learning models. Applying our proposed methodology is a simple and fast way to predict labels with class imbalance. It does not require additional techniques to balance classes. It does not involve preselecting important variables, which saves time and makes the model easy for analysis. This makes it an effective methodology for initial and further modeling of data with class imbalance. Moreover, our methodologies show how to increase the effectiveness of the machine learning models based on the standard approaches and make them more reliable.

## 1. Introduction

The topic of diabetes disease prediction has been an extremely popular topic lately. Diabetes causes an increase in the level of blood sugar, which leads to damage to various parts of the human body. Many researchers collect medical data on the physiological, social, and environmental factors that would cause diabetes. These data are used not only for providing a deeper understanding of the treatment mechanisms but also for predicting the probability that one might become sick. This prediction is important for reversing the course of the possible development of diabetes by adjusting the related factors that impact the individual. Therefore, diabetes prediction can be crucial not only for decreasing the number of cases but also for developing a better clinical approach for the individual based on the specifics of their case.

The aim of the article is to propose a novel methodology for predicting whether an individual would develop diabetes over time given a set of biological and social indicators. The proposed algorithms create effective classification models to predict the risk of diabetes. The proposed methodology works on public clinical data on diabetes in the women of the PIMA Indians dataset [1]. Our results outperform other existing research and extend the practical tools for researchers to provide a holistic approach for each patient. Based on the predictions, public healthcare specialists can create and implement specific strategies for the prevention and treatment of diabetes.

Predicting the risk of diabetes using novel computing techniques has been an expanding topic in academic literature with practical applications in medicine. Many of these techniques are centered around the application of Machine Learning (ML) models are the decision tree, support vector machines (SVM), Random Forest (RF) and Naive Bayes (NB) models [2]. For example, Traymbak et al. [3] used SVM to model the PIMA diabetes dataset and achieved a high classification accuracy of 73.86%, sensitivity of 83%, and specificity of 56.60%. In more advanced cases, deep learning techniques are applied to grasp the underlying complexity of the data and the connections among them [2]. The selection of the classification algorithm depends on the complexity of the data. The most common dataset for ML and deep learning experiments is the PIMA Indian diabetes dataset although other datasets exist as well [1]. An effective approach is to apply the ensemble and bagging methods for handling class imbalance in machine learning for healthcare datasets [4,5]. The high results emphasize the role of ensemble and bagging methods in enhancing model performance on classification models with imbalanced datasets.

For example, the RF classifier with feature selection has been applied to the PIMA diabetes dataset by Zou et al. [6]. They have concluded that the Random Forest model after the feature reduction achieves the best accuracy of 77.4% for the PIMA dataset. The same authors applied the RF model to another clinical dataset about diabetes—the Luzhou dataset. Random Forest confirms the efficiency with an accuracy value of 80.8% for the Luzhou dataset. Zhou et al. [7] have also worked on the PIMA dataset. They built an ensemble learning model based on the Boruta feature selection. The authors have applied the grid search approach to optimize the parameters of the proposed model. The accuracy they achieve is 98.0%. Machine learning models such as linear discriminant analysis (LDA), k-nearest neighbors (kNN), and Adaboost have also been applied for diabetes prediction by Traymbak et al. [3]. Different methodologies are commented on and compared with effective applications to healthcare modeling and prediction [8,9,10,11]. A detailed study on the application of SMOTE-based machine learning algorithms to predict diabetes can be found in [8,9]. In addition, Wu et al. [10] have applied machine learning modeling for imbalanced datasets based on the local interpretable model-agnostic explanation (LIME). In fact, LIME is applied to each sample independently and estimated. Then, conclusions are extended to the dataset.

Deep neural networks (DNN) are often used in complex cases. Deep neural networks are a deep learning algorithm. They combine the advantages of both deep learning and neural networks. Deep neural networks significantly increase the capabilities and quality of models applied in artificial intelligence, including diabetes prediction. Often deep learning algorithms outperform machine learning algorithms due to their flexibility and ability to capture and model more complex data structures including such in the PIMA diabetes dataset [2,12,13,14,15,16].

Regardless of the methodology selected, model estimation is a critical step in evaluating the performance of a classification model, especially when working with imbalanced data like the PIMA dataset. The standard tools for evaluating model performance are the confusion matrix and classification metrics like accuracy, precision, specificity (recall), sensitivity, and F1 score [17,18,19]. The confusion matrix provides insights into the extent to which the two classes are correctly predicted. The elements of the confusion matrix are denoted as true positive (TP), false positive (FP), true negative (TN), and false negative (FN).

The measures precision, specificity, and sensitivity can be calculated based on the elements of the confusion matrix as shown in [17,18,19]. The measures specificity and sensitivity provide information on how the model predicts both classes of the dataset. The high values of these measures confirm the efficiency of the model. The accuracy provides an insight into the overall performance of the classification models. In this study, we adhere to the standard formulas for classification evaluation [17,18,19].

Using these measures, we compare our methodology to existing ones. We show that our methodology may outperform other existing machine learning algorithms, while producing competitive results to deep learning algorithms. The advantages are simplicity, easy interpretation of the results, and improved model performance.

Next section describes the proposed methodology on the PIMA Indian diabetes dataset. The results section details the results of our experiments in terms of model quality and considering other papers on this dataset, while Section 4 concludes.

## 2. Materials and Methods

Our methodology is applied on the public PIMA Indian diabetes dataset from [1,20]. The dataset contains 768 observations and 9 variables for female patients from Arizona, USA. The dataset consists of nine medical variables (predictors) and one target variable. The target variable for the dataset represents 268 observations that are positives for diabetes. They are denoted by value ‘1’ whereas value ‘0’ is used for negative results for diabetes observations. The number of negative observations is 500. This dataset is representative of the class imbalance problem in the ML theory and practice [21,22,23]. Class imbalance is an issue in classification problems where the target variable has one class dominating over the other [23]. The structure of the PIMA dataset demonstrates heavy class imbalance as the label ‘0’ is the predominant class, accounting for about 2/3s of the observations in the target variable. Therefore, the prediction of diabetes in the PIMA dataset is sensitive to class imbalance, which needs to be handled using appropriate tools.

This paper presents a novel methodology for handling the class imbalance issue in the PIMA dataset. To interpret the results from the novel methodology, we also run experiments with the classical methodology The first methodology models the original observations without preprocessing them for class imbalance. Class imbalance is handled using the built-in parameter in Python called “class_weight” = balanced. This is a standard tool to handle class imbalnce without preprocessing the data. This is the classical approach. The second methodology introduces novel steps for data preprocessing to handle the class imbalance in the target variable before applying a classification model. The novelty in our methodology lies in resampling and shuffling the data as a preprocessing step prior to cross validation and model fitting. The classical methodology is tested with k-fold cross validation, while the proposed methodology is run by k-fold and stratified k-fold cross validation. 

K-fold cross validation is the most often used validation strategy. However, it is suitable for large datasets with no class imbalance. Some authors [16,18] argue that k-fold cross validation can be appropriate for datasets with class imbalance only if the dataset is large enough. The definition of ‘large enough’ is not provided but the PIMA dataset has less than 1000 observations, so it may be considered a ‘small’ dataset. As the definitions of ‘small’ and ‘large’ datasets are not clear, we test the proposed methodology with both k-fold cross validation and stratified k-fold cross validation. The aim is to understand which cross validation strategy is better for the PIMA dataset.

Another advantage of the proposed methodology is the unique combination of (1) resampling and shuffling, (2) setting ‘class_weight’ = ’balanced’, and (3) using (stratified) k-fold cross validation as simple steps to handle the class imbalance issue without further complicating the classification model.

**Figure 1:**
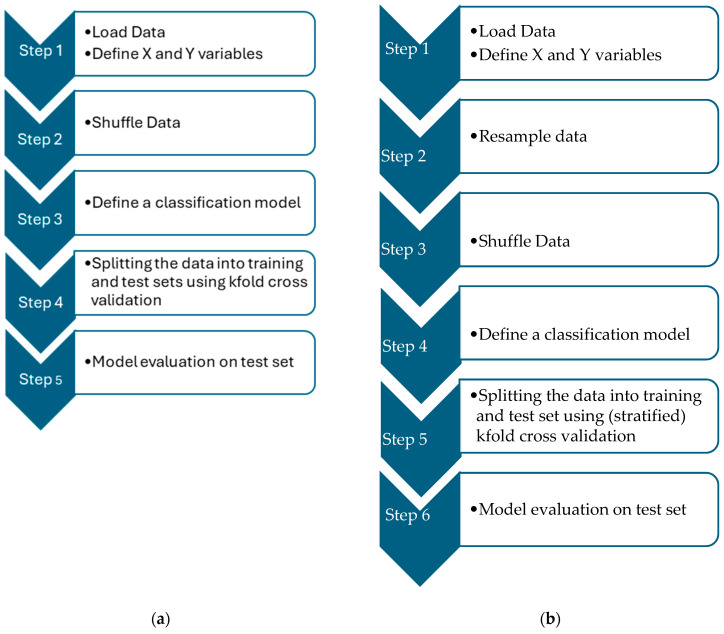
(**a**) left image summarizes Methodology 1, and (**b**) right image summarizes Methodology 2.

Figure 1 demonstrates the difference between the classical methodology and the proposed methodology.

### 2.1. Methodology 1: Algorithms 1–3—Classical Approach

Methodology 1 is presented via Algorithms 1, 2 and 3 in the investigation. The approach of Algorithms 1–3 is as follows:

Step 1: Data loading and initial processing—the initial step involves loading the dataset and delineating the independent (X) and dependent (y) variables. The y variable is transformed into categories to facilitate analysis.

Step 2: Data shuffling—the indices of all independent variables (X) and the target variable (y) have been shuffled so that their place in the dataset is rearranged. To perform the shuffle a seed of 99 is set and the numpy command np.random_permutations is used as shown below:

np.random.seed(99)

permuted_indices = np.random.permutation(len(Y))

Random shuffling in train-test splitting is employed to ensure that the training and testing datasets are representative of the overall dataset [24]. By shuffling, the data is randomized, preventing the model from learning potential patterns that may be due to the order of the data rather than the underlying relationships between the variables.

Step 3: Define two classification models—the Random Forest classifier (RF) and the support vector machines model (SVM) with parameters:

RandomForestClassifier(n_estimators = 50, max_depth = 5, random_state = 0, class_weight = ’balanced’)

SVC (C = 10, kernel = ’linear’, gamma = 0.01, probability =True, class_weight = “balanced”).

The parameter ‘class_weight’ is set to ‘balanced’ because of the class imbalance in y.

In this step the parameters as well as the type of model can be changed.

Step 4: Split the data into training and test sets using k-fold cross validation—the k-fold cross validation function is applied using different commands for classification models:

RF: KFold (n_splits = 4, shuffle = True, random_state = 555) (Algorithm 1),

SVM: KFold (n_splits = 4, shuffle = True, random_state = 763) (Algorithm 2),

SVM: KFold (n_splits = 5, shuffle = True, random_state = 673) (Algorithm 3).

Step 5. Model evaluation on the test set. The confusion matrix-model evaluation is performed using the confusion matrix and calculating accuracy, precision, specificity, and sensitivity using the formulae [17,18,19]:

$$\mathrm{Accuracy}=\frac{TP+TN}{TP+TN+FP+FN};$$

$$\mathrm{Precision}=\frac{TP}{TP+FP};$$

$$\mathrm{Sensitivity}\;(\mathrm{Recall})=\frac{TP}{TP+FN};$$

$$\mathrm{Specificity}=\frac{TN}{TN+FP}$$

Measures like specificity and sensitivity provide information on how the model predicts both classes of the dataset. The high values of these measures confirm the efficiency of the model.

### 2.2. Proposed Methodology: Algorithms 4 and 5—Classification with Data Preprocessing for Class Imbalance

This methodology contains two essential steps: (a) it applies two approaches to split training and test subsets, and (b) it applies the support vector machines model. Algorithm 4 applies k-fold cross validation to separate training and test subsets, while Algorithm 5 applies stratified k-fold cross validation for the same purpose. The approaches to Algorithms 4 and 5 are as follows:

Step 1: Data loading and initial processing—the initial step involves loading the dataset and defining the independent (X) and dependent (y) variables. The y variable is transformed into categorical labels to facilitate analysis.

Step 2: Data resampling—the second methodology applies the resampling procedure to supplement the smaller class in the set with observations. The aim is to increase their number to avoid inequality between the two classes in terms of the number of observations. The resampling function is applied with the parameters shown below:

resample(data2,replace = True, n_samples = 500, random_state = 605)

Step 3: Data shuffling—the indices of all independent variables (X) and the target variable (y) are shuffled so that their place in the dataset is rearranged. To perform the shuffle a seed of 31 is set and the numpy command np.random_permutations is used as shown below:

np.random.seed (31)

permuted_indices = np.random.permutation (len(Y))

Step 4: Define a classification model—a support vector machines classifier is defined with the parameter ‘class_weight’ set to ‘balanced’ because of the class imbalance in y. The settings of the two classifications are shown below:

SVC (C = 10, kernel = ’rbf’, gamma = ’auto’, probability =True, class_weight = “balanced”)

In this step, the parameters as well as the type of model can be changed.

Step 5: Split the data into training and test sets using k-fold cross validation—the k-fold cross validation function is applied using different commands for classification models:

SVM: KFold (n_splits = 5, shuffle = True, random_state = 42) (Algorithm 4),

SVM: KFold (n_splits = 5, shuffle = True, random_state = 73) (Algorithm 5).

Step 6: The same as Step 5 from Methodology 1.

The next section describes the output from the proposed algorithms and compares the results to others.

## 3. Results

### 3.1. Comparison of Classification Metrics

Our experiments are conducted on a laptop with 1.50 GHz Intel(R) Core (TM) and 8 GB RAM, running on Windows with Python 3.7 in the Anaconda environment. The results discuss two groups of methodologies. The first methodology is represented by Algorithms 1–3, where only a shuffling method is applied at the preprocessing stage. While the second methodology is represented by Algorithms 4 and 5 and contains a shuffling and a resampling method. To consider the output from each methodology effective, the values of accuracy, precision, sensitivity, and specificity should be high enough. The results for the classes can be averaged, so we present the average precision, sensitivity, and specificity for each algorithm.

As shown by Table 1, the first methodology results in accuracies of 83.85% (Algorithm 1), 84.9% (Algorithm 2), and 85.06% (Algorithm 3). The accuracies from the second methodology are much higher as Table 2 shows. The two Algorithms (4 and 5) in the second methodology result in accuracies of 95.5% and 90.5%. Algorithms 1–3 are considered a classical approach that underperforms when compared to the second methodology, which is a novel approach.

This finding is also visible from the precision, sensitivity, and specificity of the first methodology (Algorithms 1–3) compared to Algorithms 4 and 5. The proposed novel methodology outperforms the classical methodology when the metrics are averaged (Table 1 and Table 2). The precision for the two methodologies is similar—it varies between 90.82% and 92.56%. The high value for all algorithms shows that all of them predict correctly the positive class in more than 90% of the cases. However, methodology 1 has sensitivity scores lower than Methodology 2. The second methodology has a sensitivity of 100%, while methodology 1 achieves the highest sensitivity scores (86.54%) via Algorithm 3. As Table 1 and Table 2 show, the second methodology improved the sensitivity scores by more than 13 p.p., which is a significant improvement. A high sensitivity score shows that the model classifies correctly the observations in each class. Therefore, the proposed methodology predicts whether the patient has diabetes or not better than the classical methodology.

The second methodology also results in better specificity. Specificity demonstrates the model’s ability to correctly classify all patients that do not have diabetes. The highest score for specificity in the proposed methodology is 91.35% (Algorithm 4), whereas Algorithms 1–3 exhibit specificity scores between 82% and 86.5%. The proposed methodology improved the model’s ability to predict correctly the cases of healthy patients. The classical methodology correctly predicted healthy patients in 82% to 86.5% of the cases. While the proposed methodology captures healthy patients in more than 95% of cases.

The significant improvements in the model’s ability to predict correctly healthy patients and to identify sick patients lead to improved overall accuracy of Algorithms 4 and 5. Therefore, the finding that the proposed methodology outperforms the classical one is a result of the overall improvement of the model’s performance. The overall improvement of the model’s performance in the proposed methodology can be attributed to two factors.

The first one is handling the class imbalance issue. The classical methodology does not perform data preprocessing aimed at class imbalance. It tries to handle this issue by only setting the Python parameter ‘class_weight’ to ‘balanced’. Although this approach provides good results, our methodology offers an effective solution to the class imbalance that significantly improves the classification ability of the model. The novel steps to shuffle and resample data prior to setting the parameter ‘class_weight’ = ’balanced’ helps to get a more even distribution of the two classes so that the training/test split is undertaken in an unbiased way where the predominant class does not affect the split. Therefore, handling the class imbalance issue proves to be a key part in the quality of the model.

The second factor is the type of cross validation. As mentioned in the methods section, some authors recommend using stratified k-fold cross validation in relatively small datasets with class imbalance [23,25]. Although no explicit definition of a ‘small’ and ‘large’ dataset is given, we tested the proposed methodology with k-fold (Algorithm 4) and stratified k-fold (Algorithm 5) cross validation. As Table 2 shows, the accuracy and the rest of classification metrics are very similar in the two cases. One key finding is that the two types of cross validation can be used with this dataset, which is further proof for the lack of overfitting in our algorithms.

Another key finding is that the suitability of stratified and k-fold cross validation in target variables with class imbalance may not be defined by the size of the dataset rather than the characteristics of the data. This finding may be further explored in additional research. A third finding is that the methodology we propose is robust in terms of steps to handle the class imbalance issue. The proposed steps to handle the class imbalance issue involve shuffling and resampling data and setting the parameter ‘class_weight’ = ’balanced’. We tested these steps with k-fold and stratified k-fold cross validation and the results were similar (Table 2). This means that the novel steps we propose to handle class imbalance in the PIMA dataset are robust and are not affected by the strategy chosen for training and testing the model.

The overall quality of the models in the proposed methodology is improved and that is also confirmed by looking at the prediction for individual classes as described by confusion matrices and AUC–ROC curves.

### 3.2. Confusion Matrices and AUC–ROC Curves

The ROC–AUC (Receiver Operating Characteristic—Area Under the Curve) is another key metric for evaluating the performance of binary classification models. The ROC curve plots the true positive rate (TPR) against the false positive rate (FPR) at various threshold settings, while the AUC quantifies the overall ability of the model to distinguish between classes. Unlike accuracy, ROC–AUC is threshold-independent and remains informative even with imbalanced datasets, making it a robust tool for model comparison and selection.

Table 3, Table 4 and Table 5 show the AUC–ROC curves and confusion matrices for Algorithms 1–3, which confirm that the classical methodology performs well. High values for accuracy, precision, sensitivity, and specificity are the first indicator that the classical methodology results in correct predictions. The confusion matrix along with the AUC–ROC curve demonstrates the reliability of the standard methodology as they also confirm the quality of the model.

However, the data suffers from class imbalance. All tables related to methodology 1 (1, 3–5) show that the classical methodology performs well despite the class imbalance. But as shown in Table 2, Table 6 and Table 7 the model can perform better when the class imbalance is handled appropriately. Therefore, handling class imbalance using the proposed methodology is a better approach in the PIMA dataset.

The proposed methodology classifies the minority class more accurately, while keeping the same prediction rate for the majority class as seen by the confusion matrices of Algorithms 4 and 5 (Table 6 and Table 7). Table 6 and Table 7 show that Algorithms 4 and 5 provide the best prediction for the two classes, although the results for individual classes are close to those from Algorithms 1 to 3. Therefore, the second methodology results in more accurate predictions of the two classes, especially the minority class. This finding is key as heavy class imbalance usually leads to better prediction of the majority class and poor prediction of the minor class. As Table 6 and Table 7 show, the proposed two Algorithms (4 and 5) overcome the issue of class imbalance effectively and improve the overall quality of the model compared to the classical methodology.

### 3.3. Comparison to Other Research

As Table 2 shows, Algorithm 4 achieves an accuracy of 95.50%, an overall precision of 91.43%, an overall sensitivity of 91.35%, and an overall specificity of 100.00%. Similarly, Gupta and Goel [18] have applied several machine learning models. Their best results are obtained from the Random Forest model. The estimated parameters have the following values: the accuracy is 80.52%, the precision value is 74.47%, the sensitivity value is 72.72%, and the specificity is 90.74%. The output from the algorithms we propose outperforms Gupta’s results as seen in Table 2 and Table 8.

Table 8 also shows Tigga’s [21] and Chang’s [19] results on the PIMA dataset. Tigga’s [21] article does not apply techniques to handle class imbalance. Instead, they aim to find the most appropriate machine learning algorithm that can predict the patient’s condition correctly. Their findings suggest that the most appropriate machine learning algorithm for the PIMA dataset is the Random Forest classifier. Table 8 presents their results from the Random Forest. Compared to Gupta [18] and Chang [19], their results are worse in terms of accuracy and specificity. The second methodology we propose (Algorithms 4 and 5) outperforms Tigga’s results. A key finding in this case is that using a classifier like the Random Forest, which is known to work good with class imbalance, may not be enough to handle the bias coming from imbalanced classes

Table 8 also shows a similar case with the results of Chang [19]. They also aim to find the most appropriate machine learning model to accurately predict the PIMA dataset in the context of feature selection. They conclude that a naïve Bayes model works well when features are carefully selected, while Random Forest works better when more features are added. Although both Chang [19] and Tigga [21] conclude that the Random Forest is the most appropriate model for the PIMA dataset, Table 2 and Table 8 show that Algorithms 4 and 5, proposed in this article, outperform the Random Forest. As shown by Table 2 and Table 8, the second methodology outperforms all models used by Tigga [21] and Chang [19] (this paper presents their best results). This finding is also valid for Gupta’s [18] experiments.

The advantage of our methodology is its simplicity and fast calculation. Also, our methodology can be applied to larger or smaller datasets related to PIMA or any other diabetes dataset as we test two versions of cross validation. The k-fold cross validation, that is usually applied even in class imbalance datasets with the remark that the dataset should be large enough. We also provide a version of our methodology (Algorithm 5) with stratified k-fold cross validation, which is recommended for class imbalance in smaller datasets. Although in the case of the PIMA dataset, the type of cross validation is not the key for improved model performance, we propose two alternatives that can be the effective solution to class imbalance in other diabetes datasets with class imbalance.

Also, adding two simple steps (shuffling and resampling) before data preprocessing has proven to be an effective way to handle class imbalance as our results suggest. This finding extends the applications of shuffling and resampling in machine learning, which uncovers a new path for the discovery of new potential applications for the two of them.

We compare the results from Table 9 (based on the values of Table 7 [22]) to those obtained by Methodology 2. Table 10 presents the entries of confusion matrices when methodology 2 is applied. We calculate the percentage of the sum (FN+FP). The values are displayed in the last column of Table 9 and Table 10. Thus, the values of Table 10 are smaller than the corresponding ones in Table 9. Thus, methodology 2 minimizes the sum of false cases (FN+FP).

Further on, applying the entries of confusion matrices the values of parameters accuracy, precision, sensitivity, and specificity are computed and presented in Table 11 and Table 2.

Ejiyi and coauthors [22] also experimented with the PIMA dataset. They aim to propose a robust methodology for diabetes prediction. Their approach is different from [18,19,21] as they perform feature extraction using the Shapley Additive Explanation (SHAP). Then, they use the subset of the most important features to find the most appropriate machine learning model. They conclude that Xgboost and Adaboost perform best with SHAP. Table 11 shows their results. Their results are better than [18,19,21] as seen in Table 8. However, this is not the case when comparing the values from Table 2 and Table 11. The two tables demonstrate that methodology 2 achieves higher values of the metrics of accuracy, precision, and specificity than those obtained by Ejiyi and coauthors [22]. The measure sensitivity is 91.30%, which is slightly smaller than the corresponding value of 92.80% from Table 11. This point is another important finding as it demonstrates that combining complex techniques like SHAP, Xgboost, and Adaboost may handle class imbalance well, but a simpler and more effective methodology may still exist. The second proposed methodology is an example of a simpler and more effective methodology to predict accurately the target in the PIMA dataset, while outperforming a wide variety of existing machine learning algorithms and complex methodologies

Another advantage of the proposed methodology is that the performance is close to that of deep neural networks (DNN), which are much more flexible and reliable in capturing data anomalies. Yet, they are more complicated to use. As Table 8 shows, the accuracy of the proposed Algorithms 4 and 5 is better than that of [2,13]. The highest accuracy we achieved (Table 2) was 95.59%, while DNN achieved 98.07% [13,15,16]. The DNN accuracy is bigger than ours by about 2.5 percentage points, which is a small difference. In terms of sensitivity, the proposed methodology achieves a result of 100%, which outperforms the DNN models in Table 9, Table 10 and Table 11. For specificity, we achieve similar results to Hounguè [2].

However, classical models (Table 1) fail to handle class imbalance in a way that the prediction ability of the models does not suffer. Therefore, the results in Table 1 are not close to the results of other authors (Table 12) and the proposed methodology (Table 2). This is another evidence that handling class imbalance is a key point in modeling the PIMA dataset.

As seen in Table 2 and Table 12, other authors achieve similar results to the methodology we propose, or in some cases, slightly better results. These results are not surprising as deep learning models also capture the unstructured connections in the data and use them to train and test the model. However, machine learning models derive the boundaries between classes based on the distance among observations. Machine learning models are better at modeling structured datasets, while deep learning models capture hidden complex connections in the data.

Also, machine learning models require more involvement of the research in model tuning, while deep learning algorithms reduce the involvement of the researcher in model tuning. Therefore, deep learning models may reduce the bias coming from the researcher’s experience and knowledge when tuning the model. Deep learning models are often a good alternative to machine learning models, especially in complex datasets. Deep learning models usually perform better than machine learning models. That is why the results from the proposed methodology are remarkable.

On the one hand, the machine learning methodology we propose improves the classification ability of the model so that the results are close to complex deep learning models like neural networks. On the other hand, we achieved a specificity of 100%, a result that was not achieved by any of the deep learning models in Table 12. The proposed methodology (Algorithms 4 and 5) manages to correctly identify non-sick patients in 100% of the cases. The methodology we propose is also notable as it represents a significant improvement in the prediction ability of machine learning algorithms that become comparable to that of deep learning models. The key to this result is the steps we propose to handle class imbalance.

Another important fact is that the authors in Table 12 do not provide their confusion matrices to observe the performance of the individual classes. Confusion matrices are an essential part of the analysis of the model quality. They show whether each class is predicted correctly and to what extent. A sign for a good model is not only high classification metrics (accuracy, precision, sensitivity, and specificity) but also the correct prediction of each class. Cases when the classification metrics are high, but the confusion matrices show that one of the classes is not predicted correctly may be a sign of overfitting. Therefore, we compare our results to other authors based on the classification metrics, but the comparison of the quality of prediction for each class cannot be done thoroughly. Despite this, we achieve similar results to Table 8 and Table 12 with a much simpler methodology that is not computationally exhaustive and does not require a complex hardware setup to run. This finding presents another advantage of the proposed methodology.

Based on the results above, the key finding is that the proposed methodology is competitive with other existing machine learning and deep learning algorithms. In some cases, the proposed methodology can outperform existing ML algorithms, while having similar performance to deep learning algorithms. Resampling and shuffling the data prior to defining the classification model can improve the prediction ability of the classification in case of class imbalance. The proposed methodology can be used with either k-fold cross-validation or stratified cross-validation. In the two cases, the results are similar, which validates the importance of the two novel steps in improving the predictions for class imbalance. This result also validates the assumption made by other authors [25,26,27,28] that both k-fold and stratified k-fold cross-validation can be used in class imbalance classification. Our results also suggest that the size of the dataset in the case of class imbalance may not be the key factor determining the type of cross-validation, which opens a new field for exploration of the role of cross-validation in the class imbalance issue. The proposed methodology improves not only the classification ability of the model but the ability of the model to improve the prediction for each class. This makes it competitive with deep learning models without further complicating the applications and interpretation of the model. Therefore, we consider the proposed methodology effective and efficient in handling class the class imbalance issue for the PIMA diabetes data.

A future extension of this work would be testing the proposed methodology on larger datasets with class imbalance as well as multilabel classification. Also, the proposed methodology can be tested in datasets with many features without feature selection. We consider our methodology flexible and able to adapt to the characteristics of the data as model tuning can easily be performed. Therefore, the proposed methodology can easily be adapted to other datasets and multiclass classification.

Although some recent diabetes research focusing on various aspects of diabetes prediction exists [29,30,31,32] they use different datasets. This makes their authors’ results incomparable to ours. However, in the publication of Mohanty [33] the same dataset as ours is analyzed. The authors have studied a few machine learning models and ensemble models for classification analysis of the dataset. The proposed ensemble model in the paper [33] has achieved the following values: accuracy 84%, precision 80%, sensitivity 92%, and specificity 77% (see Table 19 [33]). Our findings with the SVM model exceed these values (see Table 2).

## 4. Discussion

In this paper we present a simple and not computationally exhaustive methodology for improving the prediction ability of classification models for the minority class in the case of class imbalance. We can summarize the key findings from this research as follows:

1. Using Python’s built-in functions can successfully tackle class imbalance. The used parameter in question is ‘class_weight’ = balance. However, additional steps need to be taken to handle class imbalance so that the model can become more efficient.
2. When having imbalanced data, resampling and shuffling the data randomly before the train/test split can help improve estimation metrics. This result is robust regardless of the type of cross validation used.
3. Applying our proposed algorithm is a simple and fast way to predict labels with class imbalance. It does not require additional techniques to balance classes. It does not involve preselecting important variables, which saves time and makes the model easy for analysis. This makes it an effective algorithm for the initial and further modeling of data with heavy class imbalance.
4. Our algorithm does not need a feature selection procedure, therefore avoiding the bias that can be introduced with the method of feature selection.
5. Two types of cross validation can be used as shown. The results are similar, suggesting that the type of cross validation may not be key for class imbalance. Rather, the overall strategy to eliminate the influence of the dominant class may be more important.
6. Despite the relatively small size of the PIMA datasets, both k-fold and stratified k-fold cross validation are appropriate. This finding contrasts with some researchers suggesting using k-fold cross validation for class imbalance only in large datasets. We highlight that the type of cross validation used may not be dependent on the size of the dataset rather than its characteristics.
7. This property of the model makes it flexible to adjust to other issues in the data, not only class imbalance. Therefore, other types of cross validation can be used.

As a conclusion, testing the proposed methodology on the PIMA diabetes dataset has shed light on the importance of resampling and shuffling data as a data processing step for handling class imbalance. With this discovery, we extend the applications of resampling and shuffling data and introduce a new line of academic research in the field of class imbalance. Moreover, our methodologies show how to increase the effectiveness of the machine learning models based on the standard approaches and make them more reliable.

Currently, the use of artificial intelligence (AI) applications in healthcare is growing rapidly. AI implementation allows healthcare professionals to devote more attention to patient care prescreening and individual treatment. As a result, the quality of life and rates of recovery increase. Therefore, the issue of the legal and ethical consequences of adopting AI technology in medicine is relevant. For example, Geantă et. al. [34] compared the efficiency of artificial intelligence models in healthcare to answer the question of which platform is better equipped to produce health-safe diagnostic models. They discovered that AI models generated by ChatGPT 3.5 in some healthcare fields may be more trustworthy and secure in diagnosing some diseases than AI models pre-defined by researchers. However, other diseases are diagnosed and treated more accurately using AI models defined by the researcher. Therefore, an ethical issue arises to discover and make sure that the safest AI algorithm for the detection of the disease is used. This results in public trust [34,35,36] in the healthcare system. However, the questions of what AI models are safe and ethical to use, how hospitals can better protect the personal data they collect as a result of more sophisticated AI models, and how can AI models be tested in reality are posed. Such scientific research supports the public debate on the use of AI in healthcare and seeks to produce new evidence in building a coherent ethical framework for AI medical technologies [36]. Therefore, proof of the ethical, clinically effective, and safe applications of each AI model should be provided before using them for diagnosis, treatment, and pre-screening [36].

Further on, in our future construction of machine learning classification methodologies for medical datasets, we will expand our research in the directions: (a) we will apply nested stratified k-fold cross-validation, (b) we will introduce additional evaluation metrics like the Matthews Correlation Coefficient, and (c) we will use the confidence intervals for applied metrics.

## Figures and Tables

**Table 1:** Results from methodology 1. Authors’ calculations.

Algorithm 1 (%) Random Forest	Algorithm 2 (%) SVM, KFOLD(N_SPLITS = 4)	Algorithm 3 (%) SVM, KFOLD(N_SPLITS = 5)
Accuracy = 83.85	Accuracy = 84.90	Accuracy = 85.06
Precision = 90.82	Precision = 92.56	Precision = 90.91
Sensitivity = 82.50	Sensitivity = 84.85	Sensitivity = 86.54
Specificity = 86.11	Specificity = 85.00	Specificity = 82.00

**Table 2:** Results from the proposed Methodology 2. Authors’ calculations.

Algorithm 4 (%)	Algorithm 5 (%)
Accuracy = 95.5	Accuracy = 95.05
Precision = 91.35	Precision = 91.74
Sensitivity = 100.00	Sensitivity = 100.00
Specificity = 91.35	Specificity = 91.00

**Table 3:** Results from Methodology 1. Random Forest, (Algorithm 1). Authors’ calculations.

Confusion Matrix	ROC Curve
[100 20] [10 62] ROC curve AUC= 90.81%	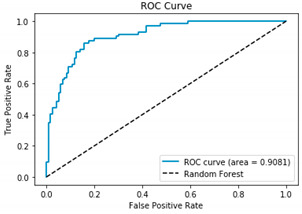

**Table 4:** Results from Methodology 1. SVM and kFold (n_splits = 4), (Algorithm 2). Authors’ calculations.

Confusion Matrix	ROC Curve
[112 20] [9 51] ROC curve AUC= 88.88%	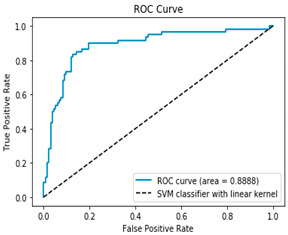

**Table 5:** Results from Methodology 1. SVM and kFold (n_splits = 5), (Algorithm 3). Authors’ calculations.

Confusion Matrix	ROC Curve
[90 14] [9 41] ROC curve AUC= 91.70%	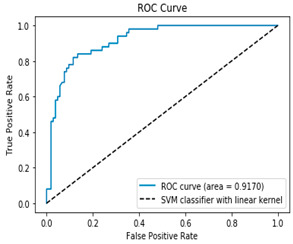

**Table 6:** Results from Methodology 2. Algorithm 4. Authors’ calculations.

Confusion Matrix	ROC Curve
[96 0] [9 95] ROC curve AUC= 97.62%	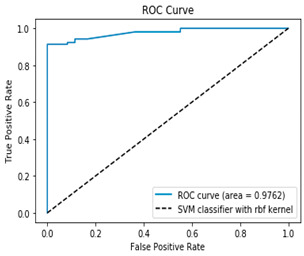

**Table 7:** Results from Methodology 2. Algorithm 5. Authors’ calculations.

Confusion Matrix	ROC Curve
[100 0] [9 91] ROC curve AUC= 96.80%	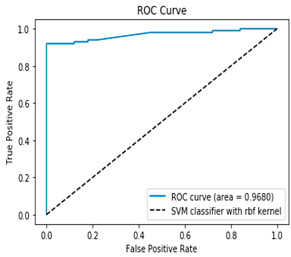

**Table 8:** Results obtained by Gupta et al. [18], Chang et al. [19], and Tigga et al. [21].

Table 6. [18] (%)	Table 3. [21] (%)	Table 13. [19] (%)
Accuracy = 80.52 Precision = 74.47 Sensitivity = 72.72 Specificity = 90.74	Accuracy = 75.0 Precision = 84.0 Sensitivity = 78.95 Specificity = 66.10	Accuracy = 79.57 Precision = 89.40 Sensitivity = 81.33 Specificity = 75.0 ROC-AUC = 86.24

**Table 9:** Entries of confusion matrices were obtained by Ejiyi and coauthors [22].

Models	TP	FN	FP	TN	Total	(FN + FP)/Total (%)
Extra Tree	141	12	18	129	300	0.1
RF	142	11	11	136	300	0.073
AdaBoost	142	11	5	142	300	0.053
GB	143	12	4	141	300	0.053

**Table 10:** Entries of confusion matrices after methodology 2. Our calculations.

Models	TP	FN	FP	TN	Total	(FN + FP)/Total (%)
Algorithm 4	95	9	0	96	200	0.045
Algorithm 5	91	9	0	100	200	0.045

**Table 11:** Results were obtained according to Table 6 [22].

Models	Accuracy (%)	Precision (%)	Sensitivity (%)	Specificity (%)
Extra Tree	90.0	88.68	92.20	87.76
RF	92.67	92.81	92.80	92.52
AdaBoost	94.67	96.60	92.80	96.60
GB	94.67	97.28	92.30	97.24

**Table 12:** Other authors obtained results via deep learning models.

Models	Measures
DNN [12] DNN [13] DNN [14] DNN + DT [15] DNN + 10-fold cross-validation [2] DNN [16]	Accuracy = 94.39% Accuracy: 98.04% Sensitivity: 98.80%. Specificity: 96.64% Accuracy: 99.4% Accuracy: 98.07% Sensitivity: 95.52% Specificity: 99.29% Accuracy: 89% Sensitivity: 87% Specificity: 91% Accuracy: 98.07%

## Data Availability

The data presented in this study are openly available here https://www.kaggle.com/uciml/pima-indians-diabetes-database (accessed on 7 October 2024) and here https://www.openml.org/search?type=data&status=active&id=43582&sort=runs (accessed on 30 December 2024).
